# Neural-enhanced motion-to-EMG: refining simulated muscle activity from musculoskeletal models using a Seq2Seq approach

**DOI:** 10.3389/fbioe.2025.1611414

**Published:** 2025-07-25

**Authors:** Tatsuya Teramae, Takamitsu Matsubara, Tomoyuki Noda, Jun Morimoto

**Affiliations:** ^1^ Department of Brain Robot Interface, Computational Neuroscience Laboratories, Advanced Telecommunications Research Institute International, Kyoto, Japan; ^2^ The Division of Information Science, Graduate School of Science and Technology, Nara Institute of Science and Technology, Nara, Japan; ^3^ Graduate School of Informatics, Kyoto University, Kyoto, Japan

**Keywords:** musculoskeletal simulation, OpenSim, muscle activity estimation, Seq2Seq with attention, spatio-temporal distortion

## Abstract

Electromyography (EMG) is essential for accurate assessment of motor function in rehabilitation, sports science, and robotics. However, its various time-consuming human operations (e.g., electromagnetic noise countermeasures) limit its widespread use. Meanwhile, motion capture technology has become more accessible, leading to increasing interest in musculoskeletal simulation models such as OpenSim. Although advances have been made in individualizing the model parameters, accurately estimating muscle activity remains a significant challenge. Previous efforts to optimize the parameters in musculoskeletal model simulators have yielded limited improvements in estimation accuracy. A key source of error that is identified in this study is the spatio-temporal distortion between the estimated and actual muscle activity, which has been inadequately addressed in previous research. To address this problem, this study proposes the Neural-Enhanced Motion-to-EMG (NEM2E) framework, which mitigates spatio-temporal distortions in simulated muscle activity using the Spatio-Temporal Distortion Refinement Network (STDR-Net). The STDR-Net is implemented via a Sequence-to-Sequence model with attention mechanisms to refine the estimates. Validation on two public datasets (walking and running motions) confirms significant accuracy improvements: enhanced estimations for all five muscles in the running dataset and for two of five muscles in the walking dataset. These findings demonstrate the potential of the NEM2E framework to refine OpenSim-generated muscle activity estimates and advance personalized applications in muscle activity analysis.

## 1 Introduction

Electromyography (EMG) is essential for accurate assessment of motor function in rehabilitation, sports science, and robotics. However, it is not suitable for routine measurement because it requires various time-consuming human operations, such as the application of electrodes by specialists and electromagnetic noise countermeasures, for correct measurement. In addition, it is affected by sweat and changes in skin conditions, making it unsuitable for long-term measurement. In contrast, markerless motion capture systems such as THEIA three-dimensional (3D), Azure Kinect, and mediapipe allow for easy motion measurement. Mobile force plates that can measure ground reaction forces simply by putting on shoes have also been investigated (Liu et al., 2010; Adachi et al., 2011) and commercialized by Tec Gihan Co., Ltd. (M3D Force Plate). In addition, VMOCAP (Ohashi et al., 2020), which estimates motion and muscle activity using only an RGB camera, has been proposed. Owing to these technological advances, muscle activity estimation using a musculoskeletal model simulator with measurements of the motions and ground reaction forces has become a useful system for measuring muscle activity in the elderly and in athletes who wish to reduce their measurement burden.

In this context, musculoskeletal model simulators (anybody, 2025; Dhaibaworks, 2025; ARMO, 2025; SIMM, 2025) such as OpenSim (Opensim, 2025; Delp et al., 2007) have attracted increasing attention for muscle activity estimation. A key challenge in musculoskeletal simulation is the accurate modeling of individual human dynamics. Optimizing model parameters such as the bone length, muscle force characteristics, and joint stiffness is crucial for achieving more realistic analyses (Dembia et al., 2021).

Among these simulators, OpenSim is one of the most widely used open-source platforms for musculoskeletal simulation (Seth et al., 2018; Mokhtarzadeh et al., 2023; Bedo et al., 2021). OpenSim facilitates the dissemination of research within the SimTK community and is used extensively in academic and clinical research (Reinbolt et al., 2011; Mansouri and Reinbolt, 2012; Lee and Umberger, 2016; Blache et al., 2017; Nasiri et al., 2022; Willson et al., 2023; Gao et al., 2024; Zhang et al., 2024). It supports integration with skeletal models (Saul et al., 2005; Christophy et al., 2012; Arnold et al., 2010) and optimization algorithms (Seth et al., 2018; Dembia et al., 2021) as add-on components. Recent advancements, such as Myosuite (Caggiano et al., 2022) combined with Multi-Joint dynamics with Contact (MuJoCo) (Todorov et al., 2012) have further expanded the capabilities of musculoskeletal simulations.

OpenSim operates through two key layers: torque estimation and muscle activity estimation (see Supplementary Appendix SA for details on the muscle activation estimation procedure). The torque estimation layer accurately predicts joint torques by customizing musculoskeletal models based on user-specific physical characteristics and ground reaction forces (Koller et al., 2018; Saul et al., 2005; Christophy et al., 2012; Arnold et al., 2010). Conversely, the muscle activity estimation layer solves an inverse problem to derive muscle activations from joint torques. However, this inverse problem lacks a unique solution owing to the redundancy of muscle actuators in the OpenSim musculoskeletal model. OpenSim addresses this challenge by minimizing the sum of muscle activity to achieve local optimal solutions (Thelen et al., 2003); however, this often leads to significant discrepancies between the estimated and measured muscle activities.

Several sources of error in the estimation of muscle activity estimation using OpenSim have been identified. Modeling errors, particularly in muscle dynamics, are one major cause. For example, prior research (Hamner and Delp, 2013) observed a consistent time delay of approximately 75 ms between the estimated and measured muscle activities in running datasets. In addition, other studies (Nasiri et al., 2022; Gastaldi et al., 2021; Liu et al., 2008) reported shifts in the peak of muscle activity. Another critical source of error arises from the non-unique solutions to the inverse problem of determining muscle activity from joint torque owing to the redundancy of muscle actuators, which are employed to reproduce the human musculoskeletal system. OpenSim uses Computed Muscle Control (CMC) (Thelen et al., 2003) to solve this problem; however, its objective function, which minimizes activation while achieving the target torques, does not guarantee alignment with actual muscle activity patterns. To the best of our knowledge, no previous study has generically addressed the motion-to-EMG problem using a neural network, as musculoskeletal simulators do.

Based on these findings, this study hypothesizes that errors in muscle activity estimation stem from two primary factors: temporal mismatches and spatial redundancy in musculoskeletal models. As the results of previous studies have shown, the resolution of these errors is limited only by the conventional optimization of the model parameters. Therefore, as opposed to optimizing the musculoskeletal parameters, this study explores an alternative approach by introducing a compensation model to refine the OpenSim outputs.

**FIGURE 1:**
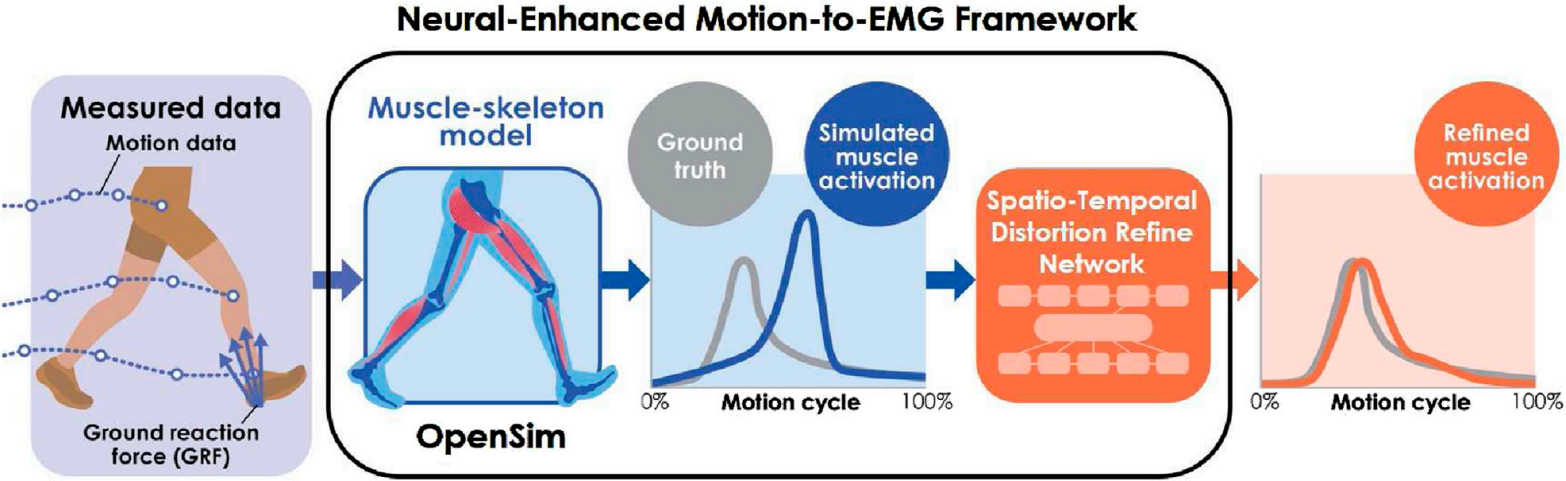
NEM2E framework. NEM2E refines the muscle activity estimated from motion and ground reaction force data using OpenSim, enhancing it with the STDR-Net.

The Neural-Enhanced Motion-to-EMG (NEM2E) framework (Figure 1) is introduced to address the above problems. It estimates realistic muscle activity from motion and ground reaction force data by incorporating the Spatio-Temporal Distortion Refinement Network (STDR-Net). Specifically, the STDR-Net refines the muscle activity outputs of OpenSim by compensating for spatio-temporal distortion. The network leverages a Sequence-to-Sequence (Seq2Seq) model (Sutskever et al., 2014), which is a recent development in natural language and image processing, enhanced with attention mechanisms (Luong et al., 2015). The framework implements Seq2Seq models with spatial and temporal attention mechanisms to investigate the spatial and temporal error contributions separately.

The analysis was performed using the walk and running motion of elderly people and athletes, as well as extractable discrete motions such as baseball batting and golf swinging, which are commonly applied in sports and rehabilitation science. This study used two publicly available datasets (Liu et al., 2008; Hamner and Delp, 2013) that pair the muscle activity estimations of OpenSim with actual EMG data for lower-limb walking and running motions. In addition, the accuracy was tested on unknown subjects through cross-validation between subjects based on open data to enable cross-sectional measurement.

The contributions of this study are summarized as follows.

• The NEM2E framework is developed to enhance the realism of musculoskeletal model simulators.
• The study hypothesizes that discrepancies between simulated muscle activity and measured EMG data are owing to spatio-temporal distortions that are inherent in conventional musculoskeletal models.
• The STDR-Net is proposed using Seq2Seq and attention mechanisms to refine the muscle activity estimations.
• Validation is performed using public datasets, demonstrating significant accuracy improvements for all five muscles in running data and two out of five muscles in walking data.

By addressing a significant error source in musculoskeletal modeling, this study opens pathways for further innovations in refining biomechanical simulations and integrating neural networks into computational modeling.

The remainder of this paper is organized as follows: Section 2 reviews related studies, Section 3 describes the NEM2E framework, Section 4 explains the model learning and statistical analysis, Section 5 presents the validation results, Section 6 discusses the findings, and Section 7 concludes the paper.

## 2 Related works

One of the key roles of OpenSim is to estimate muscle activity. Thelen et al. (2003) demonstrated that integrating joint angular acceleration feedback into the muscle activity optimization routine (static optimization) of OpenSim yields muscle activity estimates with timing similar to EMG-based measurements. This method is implemented in OpenSim as CMC.

However, estimating muscle activity from joint torque remains an ill-posed problem owing to the redundancy of actuators in musculoskeletal models. Several studies have reported discrepancies between the simulated and experimental muscle activity, which have often been attributed to changes in muscle loading and the effects of assisted movement (Nasiri et al., 2022; Gastaldi et al., 2021).

Efforts to improve the accuracy of OpenSim musculoskeletal models have followed two main approaches:

### 2.1 Development of sophisticated models

The first approach involves creating more detailed models. For instance, studies have developed precise models of the lumbar spine, addressing areas that are not adequately represented by the base model of OpenSim (Christophy et al., 2012; Raabe and Chaudhari, 2016). Tools such as NMSBuilder developed by G. Valente et al. have also been introduced to aid researchers in developing customized OpenSim models (Valente et al., 2017). However, there are inherent limitations to the accuracy that is achievable in simulations owing to challenges in replicating the complexity of the human body. In addition, measurements such as CT scans are required to capture individual differences accurately, which may not be feasible for all users.

### 2.2 Optimization of model parameters

The second approach focuses on optimizing the model parameters. OpenSim moco (Dembia et al., 2021) introduces functions to calibrate parameters such as the muscle length and maximum tension using individual user data. Although this improves the personalization, discrepancies between the estimated muscle activity and measured EMG data persist. Valente et al. proposed robust optimization methods to address uncertainties in muscle models (Valente et al., 2014). However, parameter optimization alone cannot resolve fundamental modeling errors in musculoskeletal models.

### 2.3 Our concept

In contrast to previous studies that focused only on optimizing the parameters or models within the OpenSim framework, this study introduces the NEM2E framework. By addressing temporal and spatial modeling errors separately, the STDR-Net compensates for these errors using a Seq2Seq model with an attention mechanism. This approach has the potential to mitigate the limitations of conventional methods and improve the accuracy of muscle activity estimation.

## 3 Materials and methods

This section presents an overview of the methods and experimental setup.

### 3.1 Methods

NEM2E framework and STDR-Net.

#### 3.1.1 NEM2E framework

The NEM2E framework refines the muscle activity estimations generated by OpenSim (Figure 1). It starts with the standard workflow of OpenSim, which includes 1) scaling (skeletal model parameter adjustment), 2) inverse kinematics (joint angle calculation), 3) residual reduction algorithm (joint torque estimation), and 4) CMC (muscle activity estimation) (see Supplementary Appendix SA for a detailed description). The resulting muscle activity data are then refined by STDR-Net to compensate for spatio-temporal distortions that are introduced by the OpenSim musculoskeletal model.

#### 3.1.2 STDR-Net

**FIGURE 2:**
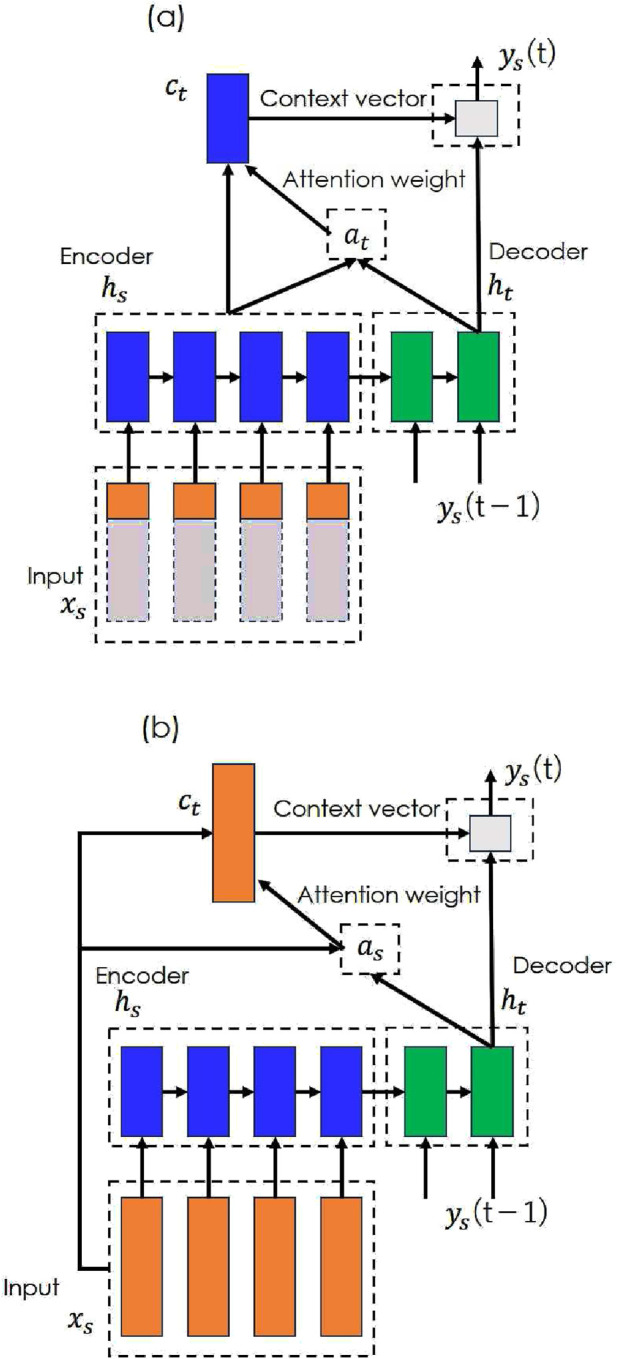
STDR-Net. STDR-Net combines the Seq2Seq model and an attention mechanism that transforms time-series data $y_{s}$ using encoders and decoders with LSTM. There are two types of attention mechanism: **(a)** an attention mechanism for temporal analysis and **(b)** an attention mechanism for spatial analysis.

STDR-Net addresses spatio-temporal distortions in OpenSim musculoskeletal models. Several time-series modeling techniques (e.g., linear regression, recurrent neural networks, long short-term memory (LSTM), and the Transformer) can be applied; however, this study implements STDR-Net using a Seq2Seq model with an attention mechanism (Luong et al., 2015) because this approach leverages attention layers to analyze the spatio-temporal relationships during data refinement. A schematic is shown in Figure 2. Two distinct models, (a) and (b), are constructed to analyze the temporal and spatial distortion, respectively.

These models use the muscle activity estimated by OpenSim as the input $x_{s}$ and the measured real muscle activity as the output $y_{s}$ , and train a neural network (encoder $h_{s}$ , decoder $h_{t}$ , and attention $a_{t}$ ) that compensates for the spatio-temporal distortions between the input and output. By analyzing the attention mechanism that is embedded in the network, the information that is used to correct the distortion can be revealed, and the contribution of spatio-temporal information to refining the estimated muscle activity can be verified. See Supplementary Appendix SB for details on the Seq2Seq with attention model.

### 3.2 Experiment

The proposed NEM2E framework was validated using two publicly available datasets (Liu et al., 2008): published in https://simtk.org/projects/mspeedwalksims and the datasets used in the literature (Hamner and Delp, 2013) published in https://simtk.org/projects/nmbl_running. The contents of the dataset are shown in Table 1 These datasets contain the estimated muscle activation results for which individual body parameter tuning and other standard tuning has already been performed in the respected studies.

**TABLE 1:** Datasets.

Dataset	Motion	Subject no.	Model	Muscle dim
Walking (Liu et al., 2008)	Four different speeds	8	gait2392	92
Running (Hamner and Delp, 2013)	Four different speeds	10	Original (Hamner et al., 2010)	102

### 3.2.1 Model learning

The parameters of the STDR-Net were trained using a dataset, where the simulated muscle activation from OpenSim served as the input and the muscle activation obtained from the measured EMG served as the output. The squared error was used as the loss function and the optimization process was carried out using the Adam algorithm (Kingma and Ba, 2015).

For all conditions, the STDR-Net was configured with 20 units corresponding to the number of muscle actuators in OpenSim for the encoder (input layer), 700 units for each intermediate layer, and 20 units for the decoder (output layer). Training was performed with a batch size of 1 and 700 epochs. The data were standardized by thinning the input and output data to 20 samples each. For the temporal attention models, the input dimension corresponded to the single dimension of the target muscle.

### 3.2.2 Statistical analysis

The performance of the proposed model was compared with the baseline muscle activity estimations of OpenSim. All EMG measurements in each dataset (running data: 16 muscles, walking data: 5 muscles) were verified. The names and abbreviations of each muscle are as follows: the soleus (soleus), biceps femoris long head (bi), semimembranosus (sem), tibialis anterior (tib), rectus femoris (rect), gluteus maximus (glmax1, glmax2, and glmax3), gluteus medius (glmed1, glmed2, and glmed3), gastrocnemius medial head (gasmed), gastrocnemius lateral head (gaslat), vastus lateralis (vaslat), and vastus medialis (vasmed) muscles. These names follow the column labels used in publicly available OpenSim muscle activity datasets and may differ from standard anatomical abbreviations. All data were segmented into individual gait cycles. A leave-one-out cross-validation approach was used, with data blocked by subject for both the walking and running datasets.

The accuracy of the model was assessed using the root mean squared error (RMSE) between the model output $\hat{y}$ and measured EMG data y , which is calculated using the following (Equation 1):

$$E=\sqrt{\frac{1}{N}\overset{N}{\underset{i=0}{\sum }}{\left(y_{i}-{\hat{y}}_{i}\right)}^{2}},$$ (1)

where N=20 is the number of samples. Assuming that the error in the refined muscle activity obtained by the proposed method is $E_{\text{pro}}$ and the error in the estimated muscle activity obtained from OpenSim is $E_{\text{OpenSim}}$ , the error improvement rate Er is calculated as

$$Er=\frac{E_{\text{pro}}}{E_{\text{OpenSim}}}-1.$$ (2)

Because each subject has data for four different speeds, the average of the four RMSE values is treated as the representative value for each subject. The null hypothesis $H_{0}:Er=0$ was tested using a T-test. The effect size is calculated using Cohen’s d in the following (Equation 3).

$$d=\frac{Er_{\text{ave}}-\mu_{0}}{Er_{\text{std}}},$$ (3)

where, $Er_{\text{ave}}$ and $Er_{\text{std}}$ denote average and standard deviation of Er , respectively. $\mu_{0}$ is hypothesized population mean.

The 95% confidence interval (CI) is calculated using the following (Equation 4).

$$CI=Er_{\text{ave}}\pm t_{\frac{\alpha }{2},n-1}\frac{Er_{\text{std}}}{\sqrt{n}},$$ (4)

where, $t_{\frac{\alpha }{2},n-1}$ represents the two-tailed t-value of $\frac{\alpha }{2}$ in the t-distribution with n-1 degrees of freedom. n is sample number. α is 0.05. Three input data patterns were defined for validation.

• One muscle, quarter cycle (One-Quarter): Sequence data for a quarter cycle of one muscle of the target muscle.
• One muscle, entire-cycle (One-Entire): Sequence data for an entire cycle of one muscle of the target muscle.
• All muscles, entire cycle (All-Entire): Sequence data for an entire cycle of all muscle sets in the musculoskeletal model.

One-Entire tested the temporal distortion compensation. Because the proposed framework emphasizes the importance of utilizing the entire sequence data to address temporal distortion, the effect of using only a portion of the sequence as input was examined in the One-Quarter model. All-Entire assessed the spatial distortion compensation by incorporating all muscle activity data. Temporal attention (Figure 2a) was used for One-Entire and One-Quarter, whereas spatial attention (Figure 2b) was applied to All-Entire. Tukey’s multiple comparison tests were performed for each muscle to analyze significant differences among the three input patterns.

## 4 Results

### 4.1 Refinement results of NEM2E

**FIGURE 3:**
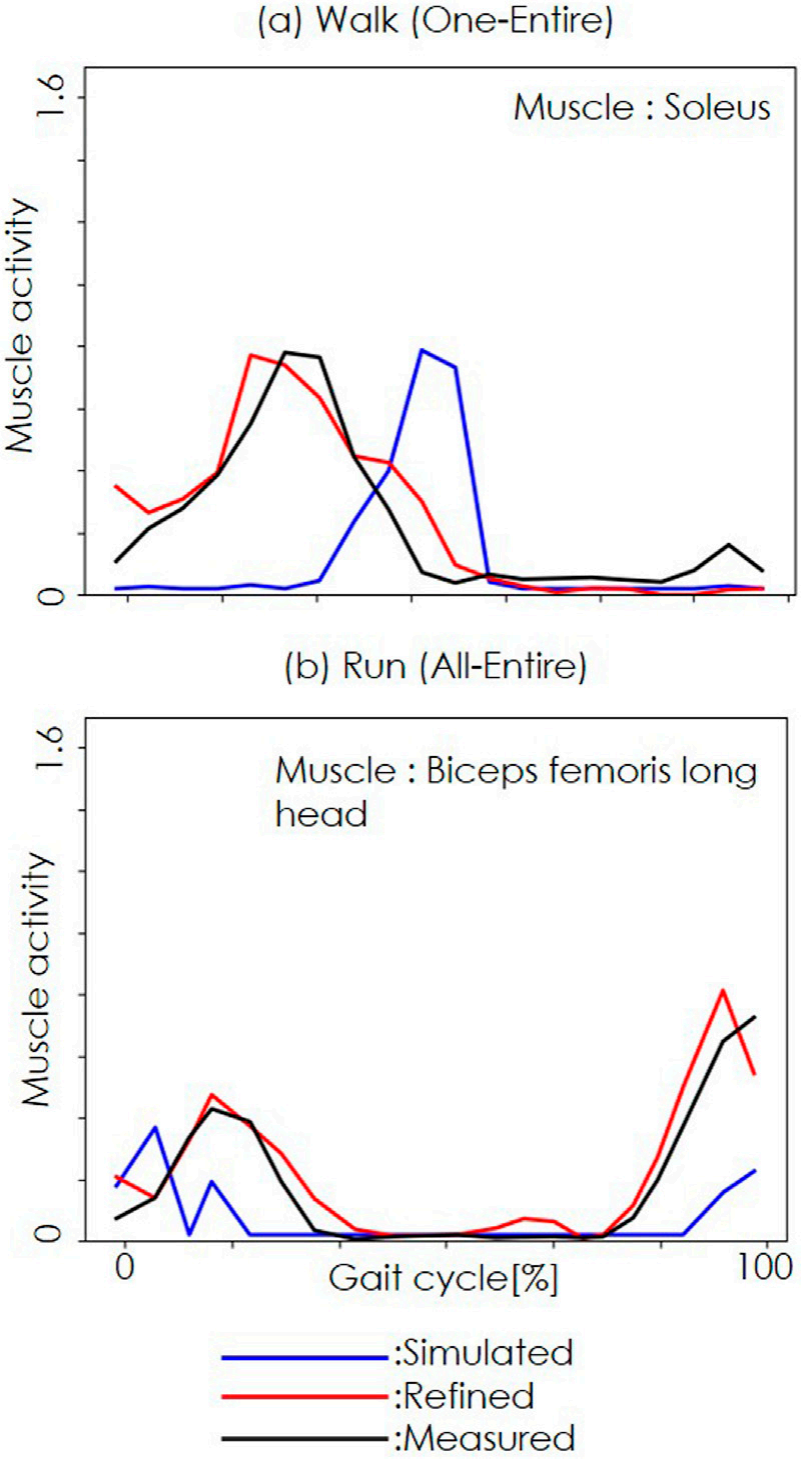
Refined muscle activities with our proposed method. **(a)** shows the walking dataset results for the One-Entire condition. **(b)** shows the running dataset results for the All-Entire condition. The blue, red, and black lines represent simulated, refined, and measured muscle activity. The horizontal axis represents the gait cycle (right heel strike to left heel strike), whereas the vertical axis represents muscle activity.

Figure 3 shows representative results from the NEM2E framework. The results for the One-Entire condition in the walking dataset are shown in (a) and the results for the All-Entire condition in the running dataset are shown in (b). The blue lines indicate simulated muscle activity from OpenSim, the red lines show the refined output from the STDR-Net, and the black lines represent measured EMG data. The horizontal axis denotes the gait cycle (right heel strike to left heel strike), whereas the vertical axis represents muscle activation. In all cases, the refined muscle activation (red) closely matched the measured EMG (black), demonstrating the ability of the framework to improve upon the baseline OpenSim estimates (blue).

### 4.2 Statistical analysis

Tables 2 and 3 summarize the statistical results for the improvement rates in the estimation accuracy for the walking and running datasets, respectively. ∗ indicates that the null hypothesis $H_{0}:Er=0$ is rejected in the estimation error improvement rate for each muscle calculated by Equation (2). In the walking data, significant improvements in accuracy were observed for the soleus, tib, and rect under the All-Entire condition. No significant improvements were observed for bi and sem under any conditions. In the running dataset, significant improvements were evident for all muscles in the All-Entire condition. Under the One-Quarter condition, only 4 out of the 16 muscles showed significant improvement, and under the One-Entire condition, 10 out of the 16 muscles demonstrated significant improvement.

**TABLE 2:** Statistical analysis for walking dataset.

	Soleus	bi	Sem	tib	Rect
One-Quarter error	−0.19 * (p = 0.036)	0.30 (p = 0.15)	**−0.034 (p = 0.57)**	−0.09 * (p = 0.015)	0.19 (p = 0.19)
95% CI	[-0.37, −0.02]	[-0.13, 0.73]	[-0.17, 0.10]	[-0.16, 0.02]	[-0.13,0.52]
Effect size, $R^{2}$	−0.92, 0.41	0.57, 0.12	−0.21, 0.22	−1.13, 0.32	0.51, 0.10
One-Entire error	−0.33 ** (p = 0.0012)	0.11 (p = 0.15)	**−0.07 (p = 0.16)**	**−0.071 (p = 0.17)**	0.19 (p = 0.26)
95% CI	[-0.48, −0.18]	[-0.05, 0.28]	[-0.18, 0.04]	[-0.18, 0.04]	[-0.17,0.55]
Effect size, *R* ^2^	−1.84, 0.56	0.57, 0.12	−0.56, 0.19	−0.55, 0.30	0.43, 0.16
All-Entire error	−0.31 * (p = 0.035)	0.15 (p = 0.062)	0.015 (p = 0.82)	−0.13 * (p = 0.025)	−0.15 * (0.033)
95% CI	[-0.60, −0.029]	[-0.0010, 0.30]	[-0.13, 0.16]	[-0.24, −0.021]	[-0.28, −0.015]
Effect size, $R^{2}$	−0.92, 0.65	0.78, 0.18	0.084, 0.12	−1.00, 0.33	−0.93, 0.28

p $<0.05{,}^{**}p<0.01{,}^{***}p<0.001$ . Bold type indicates improved accuracy.

**TABLE 3:** Statistical analysis for running dataset.

	Soleus	Bi	Semim	Tib
One-Quarter error	−0.39 ***	**−0.04 (p = 0.523)**	−0.35 ***	0.16 (p = 0.097)
95% CI	[-0.52, −0.26]	[-0.18, −0.1]	[-0.43, −0.28]	[-0.03, 0.35]
Effect size, $R^{2}$	−2.17, 0.92	−0.21, 0.77	−3.33, 0.80	0.59, 0.70
One-Entire error	−0.48 ***	−0.21 ** (p = 0.003)	−0.44 ***	**−0.03 (p = 0.68)**
95% CI	[-0.55, −0.40]	[-0.33, −0.09]	[-0.51, −0.36]	[-0.20, 0.14]
Effect size, $R^{2}$	−4.57, 0.80	−1.26, 0.44	−4.05, 0.52	−0.14, 0.38
All-Entire error	−0.59 ***	−0.42 ***	−0.47 ***	−0.14 * (p = 0.020)
95% CI	[-0.68, −0.51]	[-0.49, −0.36]	[-0.56, −0.38]	[-0.25, −0.03]
Effect size, $R^{2}$	−4.92, 0.87	−4.62, 0.59	−3.77, 0.58	−0.89, 0.40

	Rect	Semit	Glut max1	Glut max2
One-Quarter error	**−0.023 (p = 0.717)**	−0.35 ***	0.052 (p = 0.63)	0.038 (p = 0.33)
95% CI	[-0.16, 0.13]	[-0.44, −0.26]	[-0.18, 0.29]	[-0.045, 0.12]
Effect size, $R^{2}$	−0.12, 0.52	−2.80, 0.39	0.16, 0.20	0.33, 0.24
One-Entire error	−0.23 ***	−0.40 ***	0.0062 (p = 0.96)	0.0081 (p = 0.89)
95% CI	[-0.39, −0.07]	[-0.48, −0.32]	[-0.28, 0.29]	[-0.11, 0.13]
Effect size, $R^{2}$	−1.05, 0.42	−3.59, 0.47	0.016, 0.36	0.047, 0.33
All-Entire error	−0.37 ***	−0.49 ***	−0.23 ***	−0.12 * (p = 0.013)
95% CI	[-0.48, −0.25]	[-0.58, −0.40]	[-0.34, −0.13]	[-0.21, −0.032]
Effect size, $R^{2}$	−2.25, 0.52	−3.93, 0.57	−1.63, 0.39	−0.97, 0.36

	Glut max3	Glut med1	Glut med2	Glut med3
One-Quarter error	0.12 (p = 0.18)	**−0.030 (p = 0.70)**	**−0.12 (p = 0.065)**	0.044 (p = 0.58)
95% CI	[-0.065, 0.30]	[-0.20, 0.14]	[-0.25, 0.0096]	[-0.13, 0.22]
Effect size, $R^{2}$	0.46, 0.0.16	−0.13, 0.38	−0.66, 0.41	0.18, 0.40
One-Entire error	**−0.051 (p = 0.49)**	−0.23 * (p = 0.019)	**−0.027 (p = 0.80)**	**−0.10 (p = 0.33)**
95% CI	[-0.21, 0.11]	[-0.41, −0.047]	[-0.26, 0.20]	[-0.31, 0.12]
Effect size, $R^{2}$	−0.23, 0.30	−0.90, 0.45	−0.084, 0.43	−0.33, 0.53
All-Entire error	−0.18 ***	−0.35 ***	−0.29 ***	−0.23 ***
95% CI	[-0.30, −0.054]	[-0.43, −0.28]	[-0.49, −0.36]	[-0.33, −0.13]
Effect size, $R^{2}$	−1.030, 0.36	−3.32, 0.57	−0.97, 0.56	−1.59, 0.57

	Gaslad	Gasmed	Vaslat	Vasmed
One-Quarter error	**−0.097 (p = 0.33)**	**−0.096 (p = 0.20)**	**−0.037 (p = 0.60)**	**−0.030 (p = 0.70)**
95% CI	[-0.31, 0.12]	[-0.045, 0.12]	[-0.20, 0.13]	[-0.20, 0.14]
Effect size, $R^{2}$	−0.33, 0.54	0.33, 0.49	−0.17, 0.53	−0.13, 0.38
One-Entire error	−0.32 ***	−0.41 ***	−0.29 ** (p = 0.0040)	−0.47 ***
95% CI	[-0.45, −0.19]	[-0.53, −0.29]	[-0.46, −0.12]	[-0.51, −0.42]
Effect size, $R^{2}$	−1.74, 0.61	−2.41, 0.71	−1.21, 0.72	−3.59, 0.80
All-Entire error	−0.50 ***	−0.52 ***	−0.39 ***	−0.58 ***
95% CI	[-0.50, −0.29]	[-0.62, −0.42]	[-0.50, −0.29]	[-0.62, −0.42]
Effect size, $R^{2}$	−3.44, 0.74	−3.77, 0.78	−2.75, 0.80	−9.23, 0.85

*p $<0.05{,}^{**}p<0.01{,}^{***}p<0.001$ . Bold type indicates improved accuracy.

**FIGURE 4:**
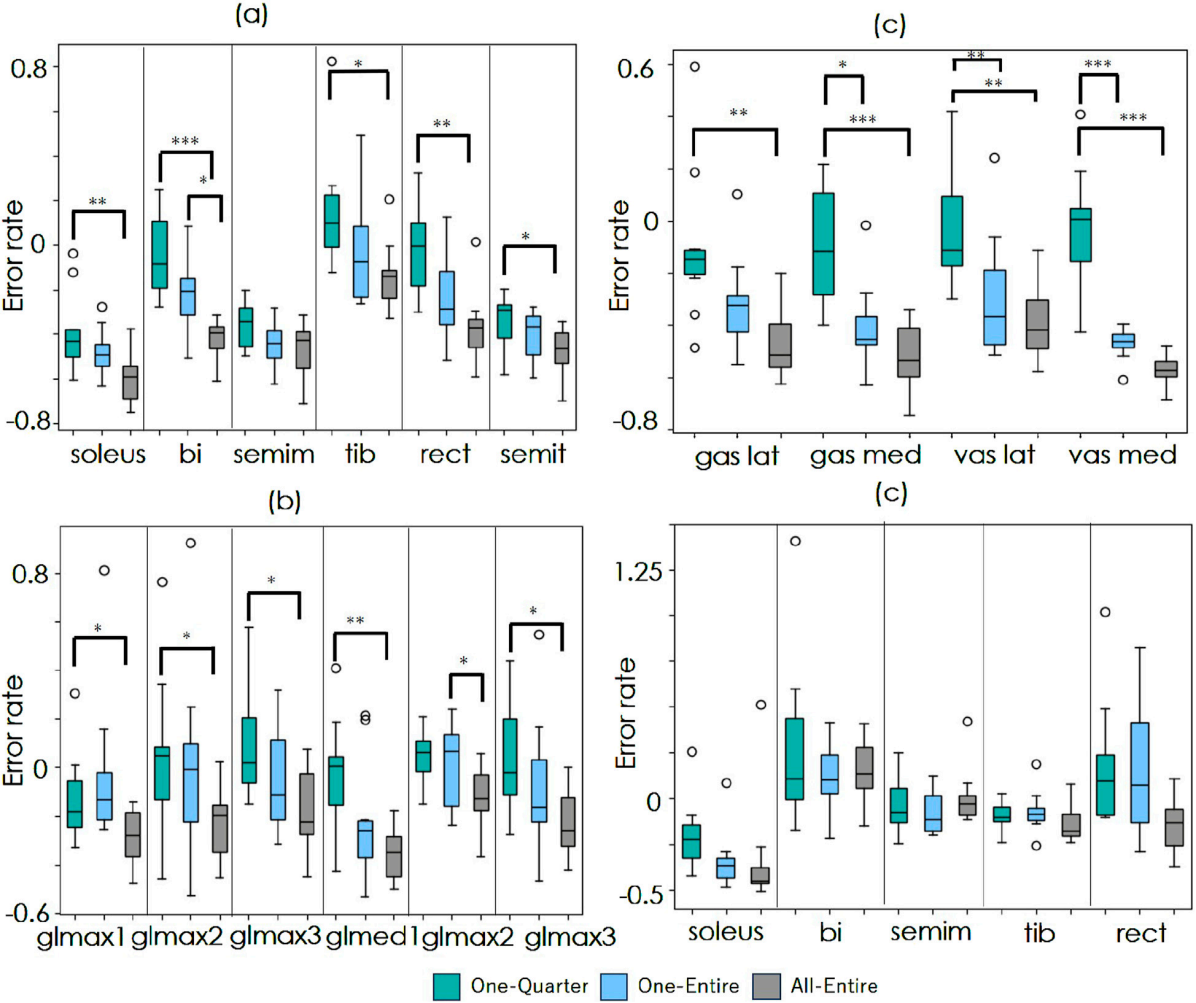
Comparison of estimation accuracy improvement. **(a–c)** show the results for the running dataset, and **(d)** shows the results for the walking dataset. The green, blue, and gray boxes represent the One-Entire, One-Quarter, and All-Entire conditions, respectively. The horizontal axis indicates target muscles. The vertical axis represents the error rate Er . Statistical significance is indicated by *p <0.05 , **p <0.01 , and ***p <0.001 (Tukey’s multiple comparisons test).

Figure 4 show the improvement rates in the estimation error for the walking and running datasets. A value of 0 represents the baseline accuracy, whereas negative values indicate improvement. In the walking dataset, there were no significant differences between conditions. Conversely, in the running data, significant differences were observed between One-Quarter and All-Entire in 13 out of 16 muscles as well as between One-Entire and All-Entire in bi and glmax2.

In the running dataset, significant improvements were observed in all muscles under the All-Entire condition. However, under the One-Entire condition, no significant improvements were observed in 6 out of the 16 muscles. Furthermore, in the walking dataset, significant improvements in the accuracy of the gastrocnemius muscle were observed under all conditions. The rectus femoris muscle showed improvements only under the All-Entire condition, suggesting that the spatial attention mechanism in this condition plays a crucial role in enhancing muscle activity estimation. Furthermore, under the One-Quarter condition, significant improvements were observed in only 5 out of 21 muscles across both running and walking data, indicating the need to consider the entire sequence rather than applying window processing to the sequence data. Moreover, as the models in this study were trained only on running and walking data, shortcut learning may have occurred, in which average muscle activity patterns were learned that are dependent on the movement duration. Then, for the One-Entire model trained on the soleus walk data, which showed strong improvement results in the validation, the improvement rate of the refined results was tested with a T-test by entering 32 randomly frequency- and phase-changed data, which was the same number of test trials in the walk dataset of the original paper. The average improvement rate (- indicates improvement and + indicates degradation) was +0.48, with a p-value of 0.001, showing significant degradation. Thus, the results demonstrate that shortcut learning, which is dependent on the operating time, did not occur.

**FIGURE 5:**
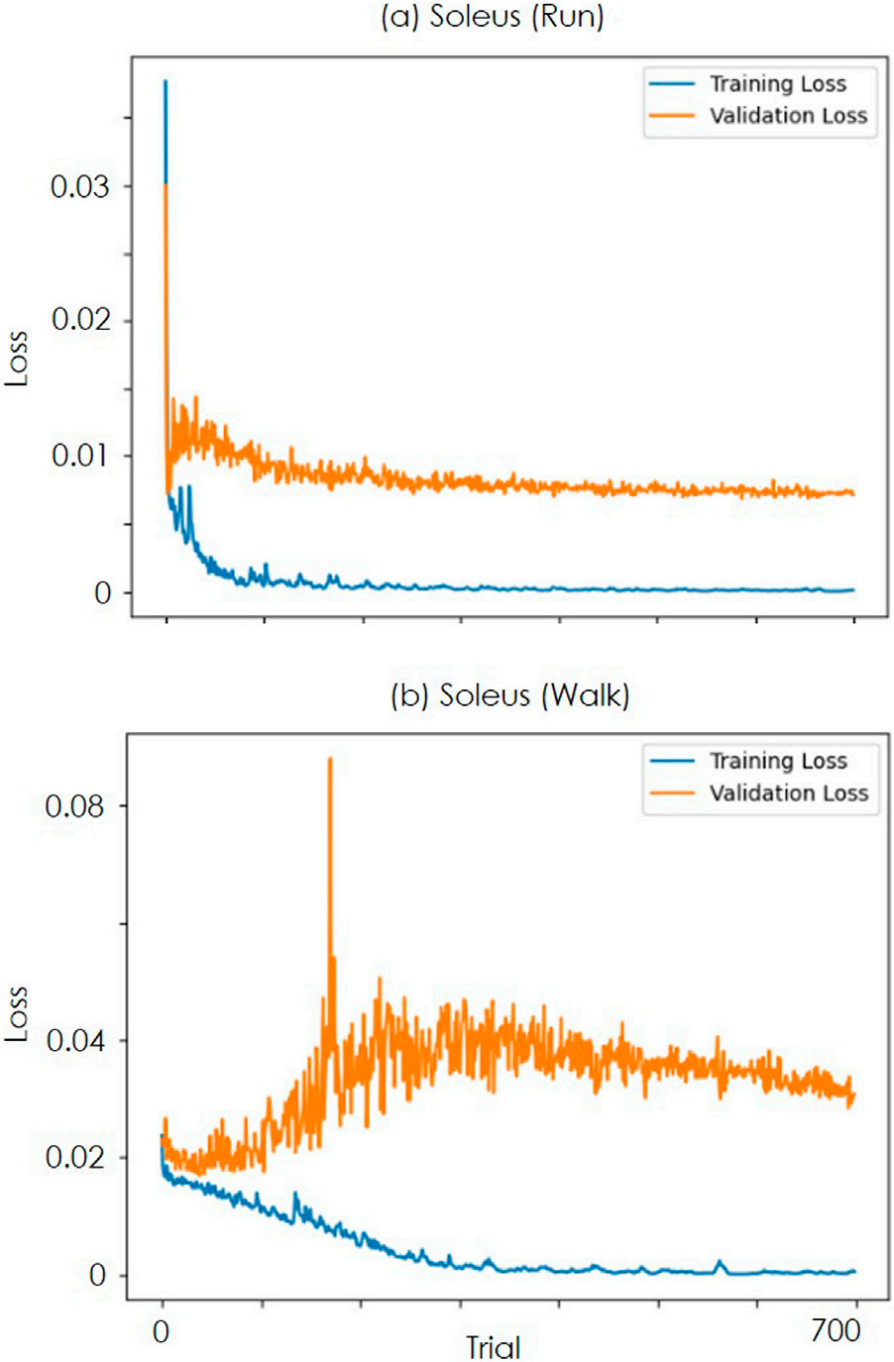
Transition of loss over trials **(a)** is the value of LOSS when learning the soleus data of the run. The blue line is the loss for the training data, and the orange line is the LOSS for the validation data. **(b)** is the result for the soleus of walking.

Furthermore, Figure 5 shows the loss trends for the training and validation data across each learning trial. In the running data, as shown in Figure 5a, the validation loss converged, whereas, in the walking data, as shown in Figure 5b, the loss temporarily increased without decreasing and then converged. For the running data, the validation loss demonstrates a stable relationship with the training loss, consistently decreasing alongside it. This trend suggests that overfitting is unlikely for this dataset, even with the relatively high number of epochs (700). The observed generalization across individuals further indicates that the model is successfully learning the underlying patterns for this specific sequence task without memorizing the training data.

Conversely, for the walk data, the validation loss exhibits an increasing trend from early epochs. However, the validation loss remains stagnant and elevated from the outset, suggesting that the intrinsic complexity or inherent noise within this dataset may hinder the model’s ability to achieve substantial learning improvements from the beginning. This observation implies that for the walk data, increasing the dataset size is considered more critical for improving model performance and generalization than simply extending the training duration or adjusting architectural parameters.

### 4.3 Attention

**FIGURE 6:**
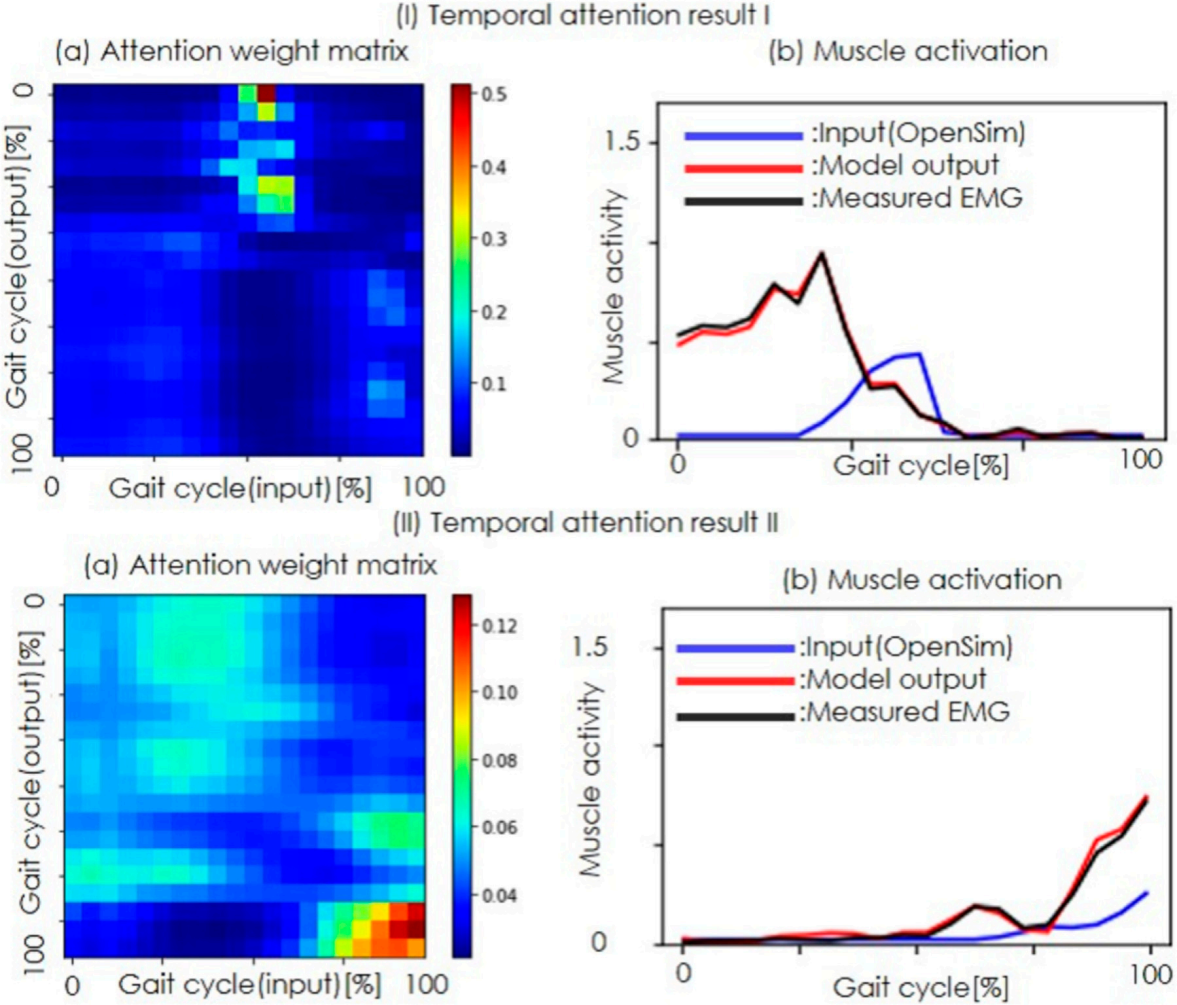
Temporal attention results. (I) and (II) show two typical examples of temporal attention. **(a)** Attention weight matrix, with the color bar indicating the weight magnitude (red for higher weights and blue for lower weights). The horizontal axis represents the input sample index and the vertical axis shows the output sample index. **(b)** Muscle activity, where the horizontal axis is the time and the vertical axis is the muscle activity. The blue lines represent the estimated muscle activity of OpenSim, the red lines indicate the refined muscle activity, and the black lines show the measured EMG.

Figure 6 depicts two representative examples of temporal attention weights in the One-Entire condition. In (I), the attention mechanism primarily focuses on the central part of the input sequence to refine the initial portion of the output waveform of the model. Conversely (II) highlights attention on the latter part of the input sequence, corresponding to the refinement of the second half of the output waveform.

The temporal attention results (Figure 6) reveal that the model allocated higher weights to data near the center of the input sequence when refining the muscle activity. This suggests that the temporal distortions in the estimations of OpenSim can be effectively learned and corrected by the attention mechanism. However, the lack of improvement in the One-Quarter condition for certain muscles, such as the biceps femoris long head, underscores the need for longer temporal sequences to capture the full distortion. The fact that the attention was focused on the peak shift in the attention weight results suggests that the model learned as intended, correcting for temporal bias rather than movement duration.

**FIGURE 7:**
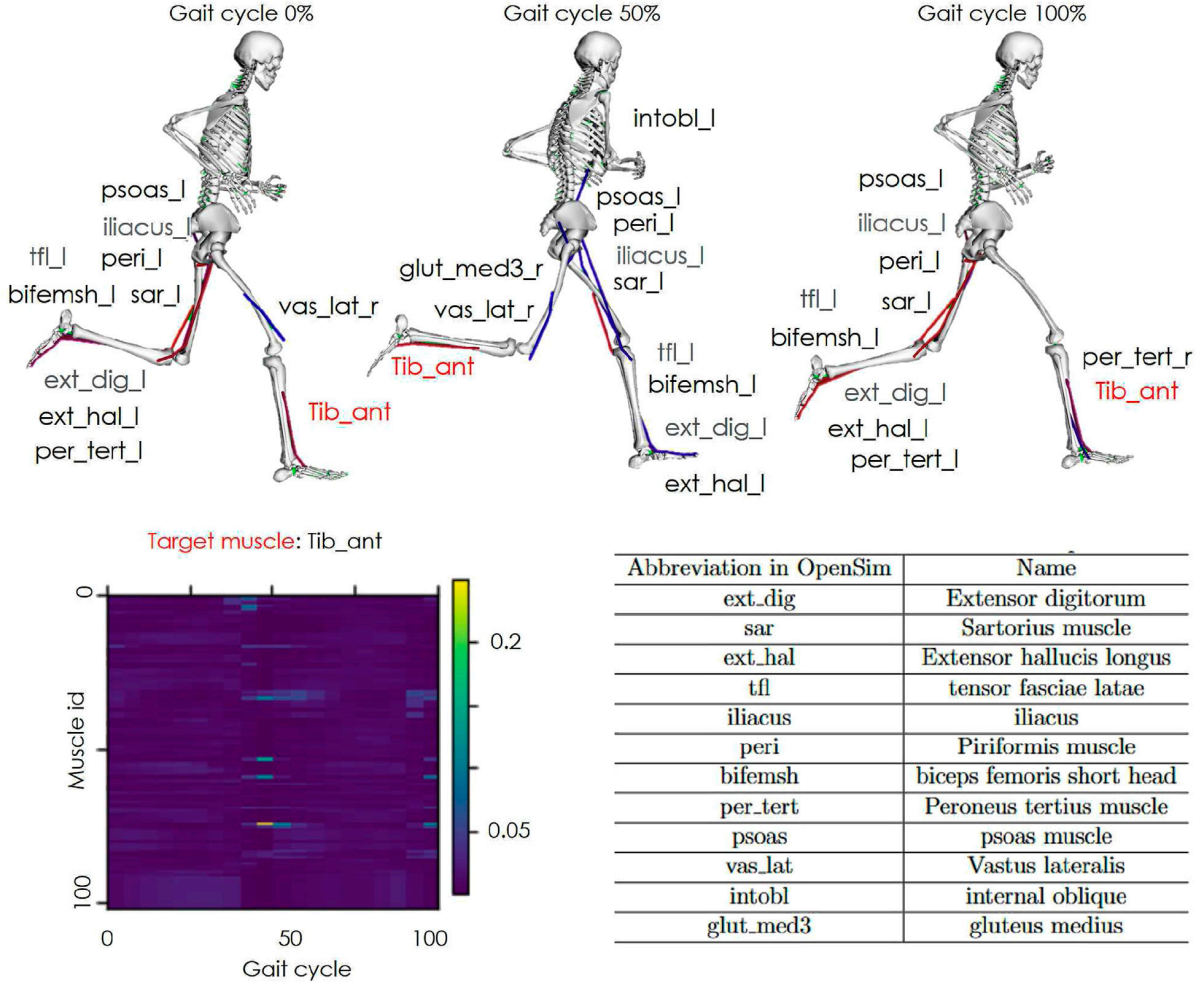
Spatial attention results. An example of spatial attention is shown. The vertical axis of the graph indicates the muscle activity channels and the horizontal axis represents the gait cycle. The color bar denotes the weight magnitude (yellow for higher weights and blue for lower weights). The skeleton models highlight the top 10 muscles with the highest motion and attention weights at 0%, 50%, and 100% of the gait cycle.

Figure 7 depicts the spatial attention results for the tibialis anterior in the All-Entire condition. Each skeleton visualizes the gait cycle motions at 0%, 50%, and 100%, with the top 10 muscles having the highest attention weights. At 0% and 100%, corresponding to the heel strike phase, similar muscle groups with high attention weights were identified. At 50%, additional trunk and hip muscles were selected, reflecting a shift in the coordination required during the swing phase.

The spatial attention revealed that the top 10 muscles with the highest attention weights for the tibialis anterior, which exhibited improved accuracy only in the All-Entire condition, varied at 0%, 50%, and 100% of the gait cycle. This variation may be explained by differences in the active muscle groups during the stance and swing phases of gait.

In addition, the top 10 muscles with the highest attention weights included muscles from the trunk, hip, and lower back on the opposite leg, in addition to the peripheral muscles of the target muscle. The peripheral muscles may have complemented the information obtained through muscle synergy during gait. Regarding the involvement of the hip, pelvis, and trunk muscles of the opposite leg, previous studies have reported that the trailing limb angle is related to ankle moments during gait (Hsiao et al., 2016). This suggests that ankle torque is not solely generated by muscle activity in the target leg, but also involves coordinated actions with the trunk and opposite leg. Owing to the significant number of muscles associated with the hip and trunk, these regions likely act as a counterweight for ankle movement. The results suggest that incorporating information from these muscles improved the accuracy of the muscle activity estimations by accounting for the broader coordination required during gait.

## 5 Discussion

We have proposed the NEM2E framework, which corrects the spatio-temporal distortion between OpenSim estimated and real muscle activity. Within this framework, a refined model for estimated muscle activity was trained using Seq2Seq with attention based on open data, and the improvement in estimation accuracy for new users was verified.

The temporal attention results shown in Figure 5a indicate that attention is focused on the 50% gait cycle muscle activity from OpenSim to estimate the 0%–40% gait cycle muscle activity. This result suggests that the proposed method refines the approximately 75 ms temporal shift observed in some muscles, as reported by a previous study (Hamner and Delp, 2013) using the running dataset. Because the temporal attention results are generalized across individuals, the results support the previous study’s suggestion that some muscles exhibit a common time delay.

In this study, we specifically address the persistent issue of spatio-temporal distortion in OpenSim muscle activity estimations. Previous studies (Koller et al., 2018; Saul et al., 2005; Christophy et al., 2012; Arnold et al., 2010) have focused on model tuning within the OpenSim environment to improve the accuracy of musculoskeletal simulators. These include tuning body parameters, aligning joint torque with floor reaction force data, and accounting for uncertainty in tuning parameters, such as muscle length. However, our proposed NEM2E framework introduces a novel external refinement approach. By treating OpenSim outputs as initial estimates and subsequently correcting their spatio-temporal properties with a dedicated refinement network, we provide a complementary solution that enhances the accuracy of existing musculoskeletal models without modifying OpenSim’s core parameters. In some cases, the improvement in accuracy could not be confirmed in certain walk data. One possible explanation is the increased variation in spatio-temporal distortions due to individual differences in balance and control strategies during the double support phase of gait. This variation may have reduced the generalization performance of the model owing to the limited dataset. Because the loss in the verification data in Figure 5b does not decrease, it is necessary to increase the size of the learning data and verify its effectiveness. Increasing the dataset size could address this issue and improve the effectiveness of the model.

A limitation of this work is the use of open data for walking and running as the validation dataset. Therefore, this method can primarily be applied to discrete motions, and the accuracy of the model for entirely unknown motions cannot be guaranteed. However, as generalization to unknown users is allowed, the method can be widely used in the analysis of movements related to walking and running, such as walking analysis of the elderly and athletics. The framework can also be applied to other discrete motions, such as golf swinging and baseball batting and pitching. However, verification regarding motions other than walking and running is crucial. In the future, it is necessary to build a benchmark dataset with a wide range of motions and large volume of data to validate the proposed framework and design appropriate refinement models.

The observed improvements in estimation accuracy for new users, particularly in generalization performance, highlights the importance of explicitly addressing spatio-temporal distortions. These findings directly support the hypothesis that spatio-temporal distortions contribute significantly to errors in OpenSim’s estimations. Furthermore, the effectiveness of the Seq2Seq with attention architecture in isolating and correcting these errors indicates that data-driven models offer a promising means of enhancing the fidelity of physiologically informed models. By externally addressing these distortions through a refinement network, the proposed framework provides a promising approach for enhancing the accuracy and applicability of musculoskeletal models. As a refined model in this study, the Seq2Seq with attention model was used as an example implementation to analyze spatio-temporal distortions; however, more suitable models may exist for this design. If a sufficient dataset can be constructed to train and compare models, the proposed framework could incorporate methods that more accuratey refine the spatio-temporal distortions discussed in this study. As a future direction, if a sufficient dataset can be constructed, research on direct estimation of muscle activity using neural network methods may become feasible.

The code to train the public dataset and perform CV between subjects under the All-Entier condition of this paper is available at SimTK (https://simtk.org/projects/nem2e).

## Data Availability

Publicly available datasets were analyzed in this study. This data can be found here: https://simtk.org/projects/mspeedwalksims https://simtk.org/projects/nmbl_running.
