# Supplementary material for: Neural-enhanced motion-to-EMG: refining simulated muscle activity from musculoskeletal models using a Seq2Seq approach
Source: Front Bioeng Biotechnol. 2025 Jul 25;13:1611414. doi: 10.3389/fbioe.2025.1611414 (PMC12331652; doi:10.3389/fbioe.2025.1611414)
Supplement: Supplementary file 1 [file Presentation1.pdf]

Neural-Enhanced Motion-to-EMG: Refining Simulated Muscle Activity from Musculoskeletal Models using a Seq2Seq Approach

S1 Appendix

Tatsuya Teramae[1], Takamitsu Matsubara[1,2], Tomoyuki Noda[1], Jun Morimoto[1,3]

1 Department of Brain Robot Interface, Computational Neuroscience Laboratories, Advanced Telecommunications Research Institute International, Soraku-gun, Kyoto, Japan
2 The Division of Information Science, Graduate School of Science and Technology, Nara Institute of Science and Technology, Nara, Japan
3 Graduate School of Informatics, Kyoto University, Kyoto, Japan

1 Estimation of muscle activation by OpenSim

The muscle activity estimation procedure in OpenSim consists of the following two layers: 1. motion-to-torque estimation and 2. torque-to-muscle activity estimation (Fig. 1). This appendix describes each of the processes that compose these two layers.

1.1 Motion-to-torque estimation

1.1.1 Scaling

Scaling involves adjusting the positional dimensions of each body, center of mass, muscle attachment positions, mass, and inertia characteristics. These adjustments are based on either a manually set scale factor or a scale factor that is calculated from the actual motion capture distance between markers. The results are used to modify dimension-dependent parameters such as the ligament and muscle lengths. In addition, static posture data are used to align the actual marker positions with those in the model.

1.1.2 Inverse Kinematics (IK)

IK optimizes the joint angles at each time point to minimize the error (Eq. (1)) between the motion capture marker tracking data and each marker position in the musculoskeletal model:

$$E_{IK} = \sum_{i=1}^{N_m} w_i(\mathbf{x}_i^s - \mathbf{x}_i^m)^2 + \sum_{j=1}^{N_s} w_j(\theta_i^s - \theta_i^m)^2, \tag{1}$$

where N_m and N_s are the total numbers of markers and joints, respectively. $\mathbf{x}^s$ and $\mathbf{x}^m$ denote the marker positions of the motion capture and musculoskeletal model, respectively, and θ^s and θ^m denote the joint angles obtained from the motion capture and musculoskeletal model, respectively. In addition, w_i and w_j are the weight coefficients.

In most cases, the relationship between the obtained joint angle trajectories and measured ground reaction force data does not match the dynamics owing to measurement, modeling, and scaling errors. Therefore, the model is optimized using the residual reduction algorithm described in the following section.

1.1.3 Residual Reduction Algorithm (RRA)

The RRA optimizes the center of mass and mass distribution of each body part using joint angular trajectories from IK and the measured ground reaction force data:

$$\min J_{RRA} = \sum_{i=1}^{N_v} (\tau_i^v)^2 + \sum_{i=1}^{N_s} w_j (\ddot{q}_j^* - \ddot{q}_j)^2, \tag{2}$$

where N_v is the number of virtual actuators, τ^v is the torque of the virtual actuators, and $\ddot{q}_j^*$ and $\ddot{q}_j$ are the angular accelerations for satisfying the joint trajectories from IK and the musculoskeletal model, respectively. OpenSim provides several solvers for solving optimization problems, but the default is the Interior Point OPTimizer.

The RRA provides optimized joint torque data for further processing.

1.2 Torque-to-muscle activity estimation

1.2.1 Computed Muscle Controls (CMC)

CMC optimizes the activity pattern of the muscle actuators to achieve the joint torque data obtained by the RRA (1). This process identifies muscle activation patterns that replicate the observed motion.

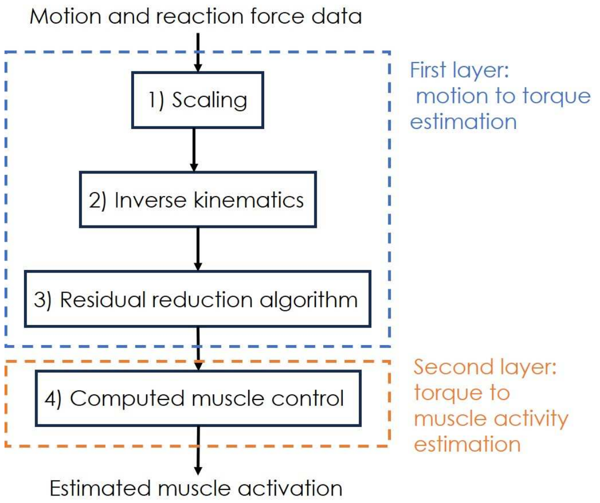

Fig 1. Flowchart of motion-to-EMG estimation by OpenSim. Opensim estimates the joint torque from motion and EMG from the torque through the following processes: 1) scaling (skeletal model parameter adjustment), 2) inverse kinematics (IK) (joint angle calculation), 3) a residual reduction algorithm (RRA) (joint torque estimation), and 4) CMC (muscle activity estimation).

References

1. Thelen DG, Anderson FC, Delp SL. Generating dynamic simulations of movement using computed muscle control. Journal of Biomechanics. 2003;36(3):321–328.
