# Supplementary material for: Neural-enhanced motion-to-EMG: refining simulated muscle activity from musculoskeletal models using a Seq2Seq approach
Source: Front Bioeng Biotechnol. 2025 Jul 25;13:1611414. doi: 10.3389/fbioe.2025.1611414 (PMC12331652; doi:10.3389/fbioe.2025.1611414)
Supplement: Supplementary file 2 [file Presentation2.pdf]

Neural-Enhanced Motion-to-EMG: Refining Simulated Muscle Activity from Musculoskeletal Models using a Seq2Seq Approach

S2 Appendix

Tatsuya Teramae[1], Takamitsu Matsubara[1,2], Tomoyuki Noda[1], Jun Morimoto[1,3]

1 Department of Brain Robot Interface, Computational Neuroscience Laboratories, Advanced Telecommunications Research Institute International, Soraku-gun, Kyoto, Japan
2 The Division of Information Science, Graduate School of Science and Technology, Nara Institute of Science and Technology, Nara, Japan
3 Graduate School of Informatics, Kyoto University, Kyoto, Japan

1 Seq2Seq with attention model

The Seq2Seq model shown in Fig. 1 comprises an encoder and a decoder. This part is common to the two different models (a) and (b) in Fig. 1. The input sequence $\mathbf{x}_s = [\mathbf{x}_s(1), \cdots, \mathbf{x}_s(T)]$ represents the simulated muscle activation and the output sequence $\mathbf{y}_s = [\mathbf{y}_s(1), \cdots, \mathbf{y}_s(T)]$ represents the refined muscle activation. The encoder $\mathbf{h_s}$ and decoder $\mathbf{h_t}$ are LSTM networks and are modeled as follows:

$$\mathbf{h}_s(t) = f(\mathbf{h}_s(t-1), \mathbf{x}_s(t)), \tag{1}$$

$$\mathbf{h}_t(t) = f(\mathbf{h}_t(t-1), \mathbf{y}_s(t-1)), \tag{2}$$

where the function $f(\cdot)$ is LSTM.

The output $\mathbf{y}_s$ is

$$\mathbf{y}_s(t) = f_y(\mathbf{h}_t(t), \mathbf{c}(\mathbf{t})), \tag{3}$$

where f_y is a fully connected layer and $\mathbf{c}$ is a context vector of attention.

Next, the attention mechanism is described. For the temporal attention mechanism in (a), the context vector $\mathbf{c}_t$ is

$$\mathbf{c}_t(t) = \sum_k^T \mathbf{a}_t(k,t)\mathbf{h}_s(k). \tag{4}$$

The attention weight $\mathbf{a}_t$ is

$$\mathbf{a}_t(k,t) = \text{softmax}(g(\mathbf{h}_s(k), \mathbf{h}_t(t-1))), \tag{5}$$

where the score function $g(\cdot)$ is defined by $g(\cdot) = \mathbf{h}_s\mathbf{W}_t\mathbf{h}_t$. $\mathbf{W}_t$ is the weight matrix.

For the spatial attention mechanism in (b), the context vector $\mathbf{c}_s$ is modeled by

$$\mathbf{c}_s(t) = \sum_k^T \mathbf{a}_s(k,t)\mathbf{x}_s(k). \tag{6}$$

The attention weight $\mathbf{a}_s$ is

$$\mathbf{a}_s(k,t) = \text{softmax}(g(\mathbf{x}_s(k), \mathbf{h}_t(t-1))), \tag{7}$$

where the score function $g(\cdot)$ is modeled by $g(\cdot) = \mathbf{x}_s\mathbf{W}_s\mathbf{h}_t$. $\mathbf{W}_s$ is the weight matrix.

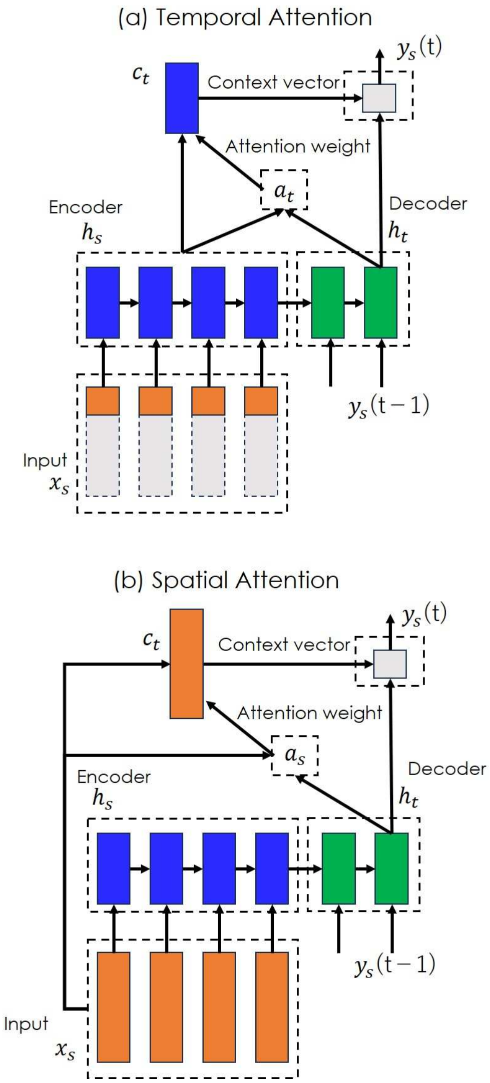

Fig 1. STDR-Net. STDR-Net combines the Seq2Seq model and an attention mechanism that transforms time-series data $\mathbf{y}_s$ using encoders and decoders with LSTM. There are two types of attention mechanism: (a) an attention mechanism for temporal analysis and (b) an attention mechanism for spatial analysis.
